# Correction: Preparation and evaluation of the biosynthetic procedure of iron oxide and magnesium oxide nanoparticles using *Hylocereus undatus* fruit peel extract and their anticancer properties

**DOI:** 10.1039/d5ra90079d

**Published:** 2025-06-18

**Authors:** Sadia Adnin Oyshi, Rumana A. Jahan, Fahima Aktar, Md. Zakir Sultan, Abu Asad Chowdhury, Jakir Ahmed Chowdhury, Shaila Kabir, Md. Shah Amran

**Affiliations:** a Department of Pharmacy, East West University Dhaka Bangladesh Sadia02021@gmail.com; b Centre for Advanced Research in Sciences (CARS), University of Dhaka Bangladesh; c Department of Pharmaceutical Chemistry, University of Dhaka Bangladesh fahima@du.ac.bd; d Department of Pharmaceutical Technology, University of Dhaka Bangladesh

## Abstract

Correction for ‘Preparation and evaluation of the biosynthetic procedure of iron oxide and magnesium oxide nanoparticles using *Hylocereus undatus* fruit peel extract and their anticancer properties’ by Sadia Adnin Oyshi *et al.*, *RSC Adv.*, 2025, **15**, 15366–15374, https://doi.org/10.1039/D4RA07411D.

The authors regret that there was an error in the equation in the ‘X-ray diffraction (XRD) analysis’ part of the ‘Experimental’ section in the original article.

The original equation: *D* = *kλβ* cos *θ*

The correct equation: *D* = *kλ*/(*β* cos *θ*)

The Royal Society of Chemistry apologises for these errors and any consequent inconvenience to authors and readers.

